# Osteomyelitis in Deep Sternal Wound Infections: Revisited—A Single-Center Observational Study

**DOI:** 10.3390/life16010008

**Published:** 2025-12-20

**Authors:** Stephan Raab, Tina Schaller, Evaldas Girdauskas, Sebastian Reindl

**Affiliations:** 1 Clinic of Cardiothoracic Surgery, University Hospital Augsburg, Faculty of Medicine, University of Augsburg, 86156 Augsburg, Germany; 2 Institute of Pathology and Molecular Diagnostics, University Hospital Augsburg, Faculty of Medicine, University of Augsburg, 86156 Augsburg, Germany

**Keywords:** deep sternal wound infection, sternum osteomyelitis, sternum osteosynthesis

## Abstract

*Objective*: Sternum osteomyelitis and deep sternal wound infection (DSWI) are often used to describe the same clinical condition interchangeably. The aim of our current study is to investigate the prevalence of osteomyelitis in cardiac surgery patients with DSWI and its consequences in therapy and osteosynthetic reconstruction. *Patients and Methods*: This is a retrospective single-center observational study. All consecutive patients with DSWI after cardiac surgery between 01/2014 and 12/2019 were included. In all patients, the sternal wound was reopened, sternal closure material was removed, and negative pressure therapy was initiated. Wound swabs were taken for microbiological examination, and a bone biopsy was examined for the presence of osteomyelitis. In the presence of osteomyelitis, long-term antibiotics were administered. *Results*: A total of 130 patients were identified in whom DSWI occurred after sternotomy. In 102 patients (77%), osteomyelitis could be detected histopathologically. The frequency of transverse sternal fractures was lower (*p* < 0.05) in the osteomyelitis subgroup (63%) as compared to the non-osteomyelitis subgroup (93%). Pathogens were detected in all patients with osteomyelitis, but less frequently (*p* < 0.05) in the group with no osteomyelitis (64%). If osteomyelitis was treated with long-term antibiotics, there was no difference in the complication rate (reinfection) after sternal restabilization between the two groups. *Conclusions*: DSWI and osteomyelitis should not be used interchangeably. If osteomyelitis can be detected histopathologically, long-term antibiotic treatment should be consistently conducted. As DSWI, with or without osteomyelitis, has been suggested to be associated with inadequate or failed sternal osteosynthesis, a key strategy to reduce its risk is to ensure safe and reliable primary sternal fixation.

## 1. Introduction

The German S3 guideline on postoperative mediastinitis after cardiac surgery presents in-depth the current state of knowledge and treatment recommendations [1]. It refers entirely to mediastinitis that occurs after a sternotomy, both after partial and complete sternotomy. However, the mediastinitis that occurs after cardiac surgery via alternative access routes, e.g., lateral mini thoracotomy, which is extremely rare, is not considered in the guideline. Incidence of surgical site infections in cardiac surgery patients has been documented to range from 3.5% to 26.8% [2]. The difference between the median sternotomy and sternum-free access routes highlights the fact that extracorporeal circulation itself is not the main cause of wound-healing disorders. Meta-analyses even state that cross-clamping and extracorporeal circulation time are longer in minimally invasive approaches as compared to median sternotomy [3]. There are two major differences between the median sternotomy and the lateral access route. In the median sternotomy, a bone is transected. Additionally, the transection of the soft tissue also differs significantly. In lateral mini thoracotomy, surgical access is always closed by means of a well-perfused muscle layer, and the skin incision is not in direct line with a bone or the surgical field. The occurrence of mediastinitis in median sternotomy obviously deals with such basic predisposing factors.

The large discrepancy in the frequency of occurrence of a DSWI highlights the fact that the definition and probably the clinical interpretation of mediastinitis are not uniform. If an infection of the sternum is assumed to be the leading cause of mediastinitis, many different definitions are used synonymously in such studies. If there is a positive microbiological result, the literature reports use the terms “surgical site infection”, mediastinitis, DSWI, and osteomyelitis almost as synonyms. This is despite the fact that many classifications exist that would clarify this [4,5,6,7,8,9,10]. Pairolero and the CDC classification differ according to the variable of osteomyelitis [5]. Jones and the Amsterdam classification differ according to sternal stability [7]. Greig and Anger differ according to the localization of the infection in relation to the sternum [8]. El Oakly and Wright differ according to the time of onset of the infection [6]. All these classifications share the same rationale that the infection of the sternum is a central element. Of note, the infection of the sternum is seldom diagnosed as secondary osteomyelitis in DSWI. Primary sternal osteomyelitis is almost always diagnosed by histopathological examination [11]. Alternatively, CT scan [12] or nuclear medical examinations [13] can be used. In the latter case, this was used in the case of suspected secondary osteomyelitis following heart surgery. The aforementioned S3 guideline also advocates a histopathological examination of the sternum with a high level of consensus; however, it provides only expert consensus as the level of evidence. Therefore, our retrospective analysis aims to address this gap of evidence in the field.

The objective of the present study was to systematically assess the prevalence of concomitant osteomyelitis in cardiac surgery patients presenting with DSWI as well as the associated pathogen spectrum through histological and microbiological evaluation. In addition, the study aimed to examine the implications of these findings for therapeutic management and subsequent osteosynthetic reconstruction.

## 2. Patients and Methods

A retrospective analysis of all consecutive patients who underwent cardiac surgery using extracorporeal circulation with median sternotomy between 01/2014 and 12/2019 was performed. Only wiring had been used for the primary closure of the sternum. All patients with DSWI were identified from this population. All patients had DSWI in the postoperative phase. This was diagnosed based on the following criteria:

- Signs of local infection (e.g., redness, heat, swelling, and pain)
- Palpatoric instability of the primary osteosynthesis (wire cerclages)
- Abnormal or putrid wound secretion
- Elevated inflammation markers (CRP and leukocyte count)

Based on the clinical suspected diagnosis, a thoracic CT scan was optionally performed to evaluate the extent of the wound infection, particularly regarding mediastinal involvement, as well as to assess a possible disruption of the wire cerclages. The following surgical procedure was then performed on these patients: the sternal wound was completely reopened. As part of the surgical wound debridement and removal of necrotic tissue, specimens were obtained for microbiological examination. A negative pressure system was then inserted and changed at 3-day intervals. Microbiological samples were obtained repeatedly to monitor infection eradication. Before re-osteosynthesis, a bone sample was taken by means of the oscillating saw. For this purpose, a thin bone lamella was resected from each sternal half, both to create smooth wound margins and allow optimal anatomical repositioning, as well as to obtain tissue for subsequent histopathological analysis. These were examined for the presence of osteomyelitis. Re-osteosynthesis was then performed. All osteosyntheses were covered with a bilateral pectoralis plasty. The follow-up period of the whole study cohort was 12 months. If a skin fistula with secretion as a sign of chronic osteomyelitis or recurrent clinical signs of reinfection occurred in the further course, bone healing was monitored for 6 months. A repeat swab was obtained, and antibiotic treatment was maintained for at least 3 months according to the bacterial spectrum in accordance with the treatment of osteomyelitis. The osteosynthetic material was then removed subsequently and the wound was closed again.

In addition to the usual patient parameters, the following variables were recorded for the purpose of this study, according to available patient records, microbiological and histopathological findings, as well as surgical reports from the primary operation, all dressing changes/wound debridements, and the surgical stabilization of the sternum. Complete data were recorded for all patients, including:

- Time of occurrence of the primary wound healing disorder
- Sternum transverse fractures as a sign of tearing of wires through the bone
- Histologically confirmed osteomyelitis
- Bacteriological spectrum of intraoperative microbiological samples
- Occurrence of delirium

All quantitative data were collected and analyzed using SPSS V31, IBM, New York. Statistical tests included the Mann–Whitney U-test, Fisher’s exact test, and χ^2^-test; statistical significance was assumed at a *p*-value < 0.05.

## 3. Results

**Figure 1:**
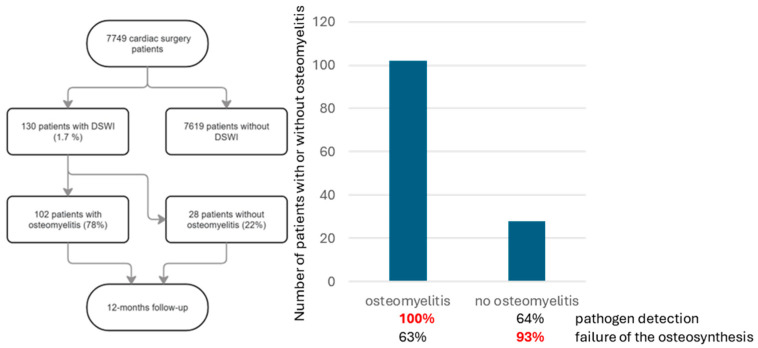
Flowchart for patient selection and follow-up. Of 130 patients with DSWI, 103 had osteomyelitis.

**Figure 2:**
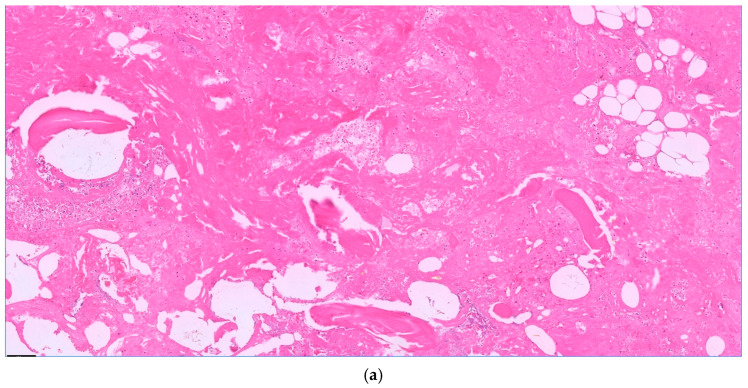

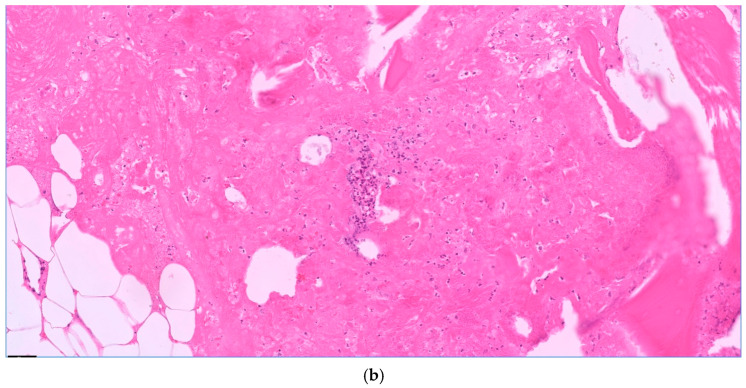
Acute osteomyelitis with granulocyte aggregates, necrosis, and fibrin. Bone balls without nuclei as a sign of necrosis. H.E. staining, magnification 10× (**a**) and 20× (**b**).

**Figure 3:**
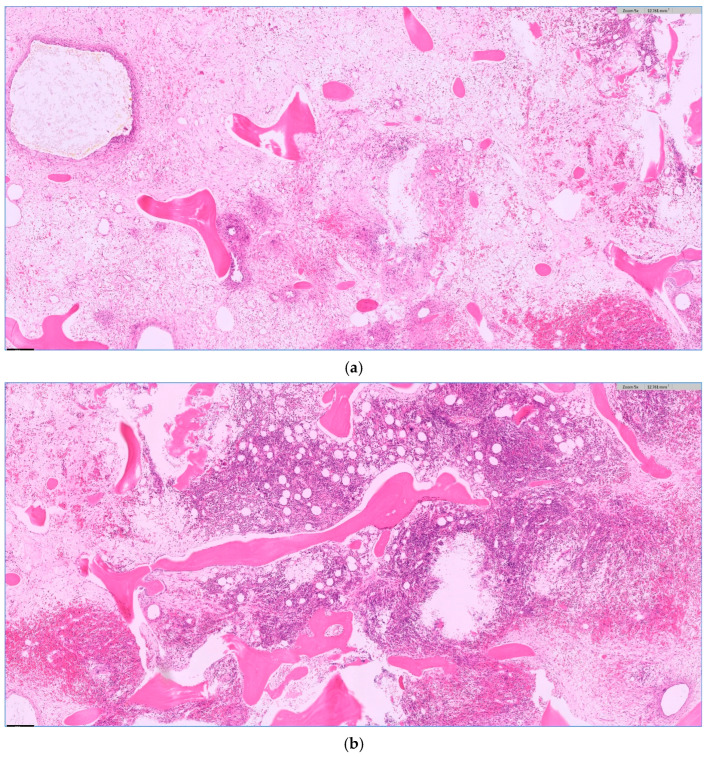
Chronic osteomyelitis with fibrosis (**a**) and granulation tissue (**b**) as a sign of a prolonged change. (**a**,**b**) H.E. staining, magnification 5×.

During the study period (i.e., 01/2014 to 12/2019), 7794 patients underwent cardiac surgery via median sternotomy using extracorporeal circulation at our institution. A total of 130 (1.7%) patients developed a DSWI and underwent the standardized treatment, as described above (Figure 1). Data on these patients are shown in Table 1. Osteomyelitis could be confirmed histopathologically in 102 patients (77%) (see Figure 2a,b for acute, and Figure 3a,b for chronic osteomyelitis). Thus, the remaining 22% of patients had no evidence of osteomyelitis. The frequency of transverse fractures, due to sternal wires being pulled through the sternum, was 63% in the osteomyelitis subgroup as compared to 93% patients in the non-osteomyelitis cohort. This is also statistically significant, *p* < 0.05 (χ^2^-test).

Subgroups with osteomyelitis vs. non-osteomyelitis did not differ significantly in risk factors and comorbidities. Both groups were comparable in age (67.9 ± 9.7 vs. 67.8 ± 9.4 years; *p* = 0.840) and showed a high prevalence of obesity (81.7% vs. 82.1%; *p* = 1.00; OR = 0.97) with a BMI of 29.33 ± 4.65 vs. 30.82 ± 5.32 kg/m^2^ (*p* = 0.243). Prevalence of diabetes mellitus (35.3% vs. 35.7%; *p* = 0.964; OR = 0.98) and anamnesis of smoking (55.6% vs. 66.7%; *p* = 0.33; OR = 0.63) were not statistically different in the osteomyelitis and the non-osteomyelitis subgroups. The occurrence of postoperative delirium had no influence on the occurrence of osteomyelitis as well. In the osteomyelitis subgroup, 66% had postoperative delirium, and in the subgroup without osteomyelitis, 70%. Overall mortality was numerically higher in patients with osteomyelitis than in the osteomyelitis subgroup (7.8% vs. 3.6%); nevertheless, the difference was not statistically significant (Fisher’s exact test: *p* = 0.67; OR = 2.30; 95%-KI: 0.27–19.20).

**Figure 4:**
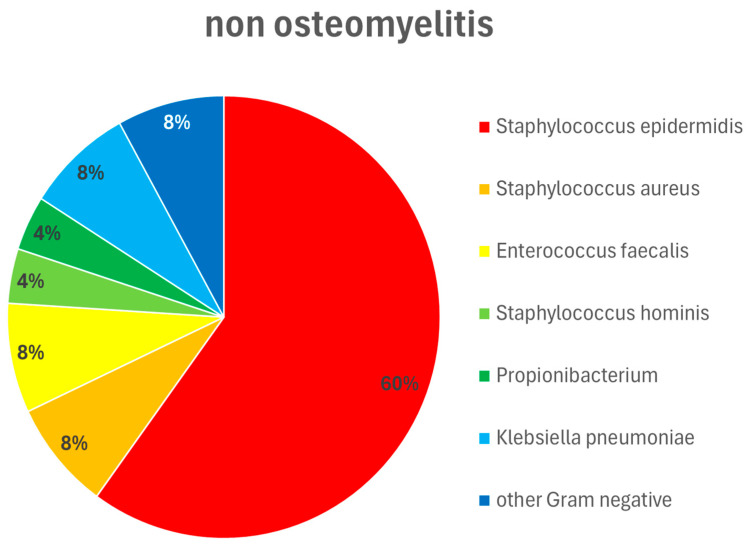
Pathogen type and distribution in patients without osteomyelitis. Wound swabs were obtained intraoperatively during wound debridements and dressings. Staphylococcus epidermidis 60%, Staphylococcus aureus 8%, Enterococcus faecalis 8%, Staphylococcus hominis 4%, Propionibacterium 4%, Klebsiella pneumoniae 8%, other Gram-negative pathogens 8%.

**Figure 5:**
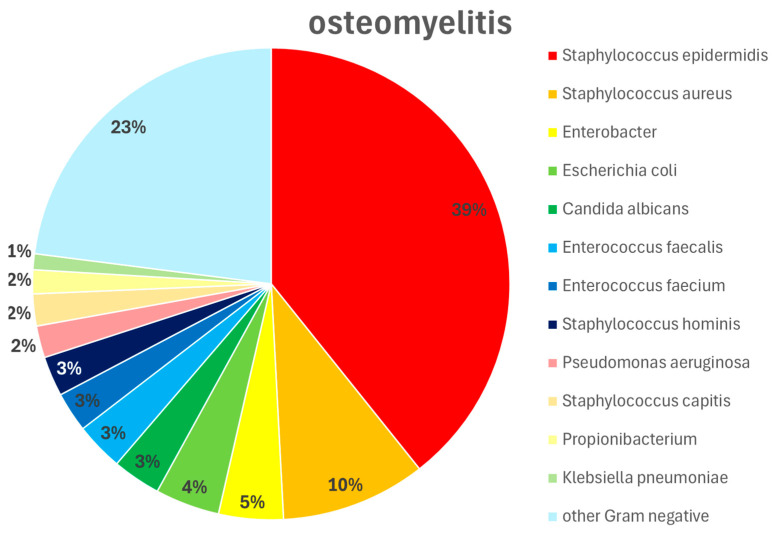
Pathogen type and distribution in patients with osteomyelitis. Wound swabs were obtained intraoperatively during wound debridements and dressings. Staphylococcus epidermidis 39%, Staphylococcus aureus 10%, Enterobacter 5%, Escherichia coli 4%, Candida albicans 3%, Enterococcus faecalis 3%, Enterococcus faecium 3%, Staphylococcus hominis 3%, Pseudomonas aeruginosa 2%, Staphylococcus capitis 2%, Propionibacterium 2%, Klebsiella pneumoniae 1%, other Gram-negative pathogens 23%.

Regarding the time of occurrence of a DSWI, there was no difference between the two subgroups (i.e., 18.8 +/− 15.4 days vs. 18.9 +/− 16.6 days postoperatively, respectively). On the other hand, there was a significant difference (*p* < 0.05) between the groups with and without osteomyelitis regarding the presence of pathogens in the wounds. In patients with osteomyelitis, a pathogenic microorganism could always be detected. In patients without osteomyelitis, this was only possible in 64% of cases. There were also differences in the bacterial spectrum. These are summarized in Figure 4 and Figure 5. Regardless of the presence of osteomyelitis, the wounds were infected with bacteria in both groups. However, there was no difference between the groups in the frequency of successful eradication of the bacteria from the wound. Overall, 42.8% of the wounds in patients with osteomyelitis were still colonized with bacteria at the time point of sternal re-stabilization and wound closure. In patients without osteomyelitis, the prevalence of wound colonization was 38.2%. In both patient groups, this was almost exclusively Staphylococcus epidermidis.

Infectious complications after refixation were analyzed during the follow-up. There was no significant difference between the two groups (χ^2^-test, *p* = 0.93). In the group with osteomyelitis, 28.4% had an infectious complication, and in patients without osteomyelitis, 21.4%.

## 4. Discussion

Deep sternal wound infection is not a uniform disease pattern. The results of this study suggest that two subgroups can be formed: an osteomyelitis group and a non-osteomyelitis group. In patients without osteomyelitis, it is noticeable that they have significantly more transverse fractures of the sternum. On the other hand, a bacterial pathogen is significantly less frequently detected in these patients when the wound is opened. Based on the presented data, two different ways of developing DSWI can be postulated. The first is initiated by a pathogen-mediated infection, which is a prerequisite for the development of an osteomyelitis, even with negative microbiological cultures. The second pathway originates from primary mechanical instability. Owing to the sternum’s anatomical position—immediately subcutaneous and covered by only a thin soft-tissue envelope—even superficial infections have the potential to extend into the bone and progress to subsequent osteomyelitis. The latter represents a distinct group of DSWI patients. In such patients, sternal osteosynthesis fails due to very high stress or due to the fact that osteosynthesis is inadequate for the patient. Therefore, the prevention of a DSWI requires adequate and safe osteosynthesis.

The findings for the second subgroup, which presented with infection and osteomyelitis, are discussed in the following section. External contamination intra- or postoperatively has been shown to be a minor factor. Previous studies revealed that DSWI is predominantly an endogenous development [14]. The predisposing factor is likely to be the infection of a hematoma adjacent to the sternum. In this regard, there exist obvious parallels to the surgery dealing almost exclusively with osteosynthesis and bone prostheses. In orthopedic surgery and trauma surgery, a hematoma on the osteosynthetic material or prosthesis is well-known to pose a high risk of infection. A Finnish registry study investigated the frequency of infection after knee replacement: 484 infections occurred in 6087 patients. Intraoperative bleeding > 100 mL was identified with a hazard ratio of HR 1.7 [15]. This finding was later confirmed in several studies and meta-analyses. Consequently, tranexamic acid is used topically in prosthesis surgery. A meta-analysis of more than 2 million cases following hip and knee replacement is cited here as the basis for this finding. It was shown that the use of tranexamic acid reduces the incidence of infection by 1% (RD = −0.0095 [95% CI −0.013 to −0.005], *p* < 0.001) [16].

If a hematoma is supposed to promote sternal infection, this can be triggered by two mechanisms: either by even small movements at the osteosynthesis or by exposed bone marrow. Both pathways can be prevented by adequate osteosynthesis for the patient. Both cortices of the sternum must match and be fixed with an osteosynthesis that can also withstand shear forces. In this respect, the topical application of vancomycin in the sternal cleft during osteosynthesis has been suggested [17]. It is not only the potential benefit of topical antibiotics that seems to have an effect here [18], but also the reliable coverage of the trabecular bone to avoid the development of a hematoma. In view of the broad spectrum of pathogens in DSWI with and without osteomyelitis (see Figure 4 and Figure 5), antibiotic therapy should be guided by multidisciplinary teams involving an infectious disease specialist, following the principles of antibiotic stewardship. Similar to orthopedic surgery, tranexamic acid may also be considered for a topical application. However, there is no published study on this approach in cardiac surgery.

Our data showed that osteomyelitis can be detected histologically in over ¾ of all cases of DSWI. This also justifies to a certain extent that the terms osteomyelitis, deep sternal wound infection, and mediastinitis are mixed together in studies without histological evidence of osteomyelitis. Postoperative imaging is essential for evaluating complications after median sternotomy. This includes osteomyelitis, abscess formation, cortical destruction and inflammatory reactions of the periosteum. Magnetic resonance imaging (MRI) is considered the gold standard for diagnosing sternal osteomyelitis due to its soft-tissue contrast and ability to detect bone marrow involvement. However, the presence of metallic sternal wires can generate artifacts that may limit diagnostic accuracy [19]. Other detection methods, such as CT scan [12] and nuclear medicine examinations, in particular scintigraphy [13] as single photon emission computed tomography (SPECT) with 99mTc-hexamethylpropylene amine oxime (HMPAO)-labeled leukocytes, are justified for the diagnosis of osteomyelitis before the wound is reopened.

Thus, a distinction can also be made between a superficial infection and osteomyelitis. If osteomyelitis is detected, antibiotic treatment should be administered in accordance with the guidelines for osteomyelitis. Conversely, the exclusion of osteomyelitis can prevent this prolonged antibiotic treatment. This approach is supported in this study: With effective osteomyelitis treatment with antibiotics for 6 weeks, the rate of recurrence of complications after sternal refixation is not statistically significantly different from the group without osteomyelitis.

Radical resection of the sternum [20], as some authors recommend, seems to be obsolete in the absence of osteomyelitis. The best procedure here is negative pressure therapy [21] with subsequent sternal stabilization as described in this study.

The presented results are based on single-center retrospective data and therefore have limitations. Diagnosis of DSWI was determined clinically and intraoperatively. Thoracic imaging was not acquired systematically. However, the subject of this analysis was not the overall concept of treating a DSWI, but rather a special attention to the secondary osteomyelitis after sternotomy. It describes how this can be reliably diagnosed and what the consequences are regarding prophylaxis and treatment. Although this is a retrospective analysis of a single center, it is the first description of the histological processing of sternal samples before re-stabilization and can therefore support the expert consensus of the S3 guideline on mediastinitis.

## 5. Conclusions

A DSWI should not be equated with a diagnosis of osteomyelitis. Therefore, in addition to microbiological examination, consistent histopathological processing of sternal samples is necessary. This identifies patients who require long-term antibiotics. Both overtreatment and undertreatment with antibiotics can thus be avoided.

## Figures and Tables

**Table 1:** Data of patients with DSWI.

Patient Characteristics	Value
age (years)	67 +/− 15.2
gender (male:female)	6.1:1
obesity	109 (84)
BMI	29.5 +/− 4.69
diabetes mellitus	46 (35)
smoker anamnesis	66 (51)
CABG	84 (64)
CABG + AVR	14 (11)
ASD II closure	2 (2)
MVR	1 (1)
AVR	13 (10)
MV repair	2 (2)
MV repair + TV repair	1 (1)
AAR	12 (9)
AVR + AAR	1 (1)
OR time (skin-to-skin) (min)	183 +/− 46.8
median sternotomy	129 (99)
partial sternotomy	1 (1)
length of ICU-stay (days)	8.05 +/− 12.01
length of ICU-stay > 48 h	56.9 (44)
postoperative delirium	53 (41)

Data presented as absolute numbers and percentages. Obesity was defined as a body mass index (BMI) exceeding 30. The history of diabetes mellitus and smoking status were extracted from the patient’s medical record. OR Operation Room, ICU Intensive Care Unit.

## Data Availability

The data presented in this study are available on request from the corresponding author.

## Abbreviations

The following abbreviations are used in this manuscript:

DSWI	Deep Sternal Wound Infection
CT	Computed Tomography
CRP	C-reactive protein
SPECT	Single-photon emission computed tomography with 99mTc-hexamethylpropylene Amine oxime (HMPAO)-labeled leukocytes
H.E.	Haematoxylin–eosin staining
CABG	Coronary artery bypass grafting
AAR	Ascending aortic replacement
ASD	Atrial septum defect
AVR	Aortic valve replacement
MV	Mitral valve
MVR	Mitral valve replacement
TV	Tricuspid valve
